# INTERACTION OF SPERMIDINE AND A LOW-METHIONINE DIET ON LIFESPAN IN MTDNA-DEFICIENT SACCHAROMYCES CEREVISIAE

**DOI:** 10.1093/geroni/igae098.2293

**Published:** 2024-12-31

**Authors:** Wei-Hsuan Su, Evien Cheng, Laura Hernandez Cervantes, Megan Nishitani, Samuel Schriner

**Affiliations:** University of California Irvine, Irvine, California, United States; University of California Irvine, Irvine, California, United States; University of California Irvine, Irvine, California, United States; University of California Irvine, Irvine, California, United States; University of California Irvine, Irvine, California, United States

## Abstract

Spermidine and a low-methionine diet are two strategies shown to extend lifespan in model organisms, such as yeast, worms, flies, and rodents. Mitochondrial dysfunction is one of the theories explaining how organisms age. In previous studies, we used yeast lacking mtDNA (rho0) as a model for mitochondrial dysfunction. We found that spermidine was not only unable to extend lifespan in rho0 yeast, but actually shortened lifespan. Spermidine and methionine are closely linked metabolically as S-adenosylmethionine from the methionine cycle can be used to synthesize spermidine. And in turn, 5’-methylthioadenosine from the conversion of spermidine to spermine can be used to synthesize methionine. While we found that low-methionine treatment could extend wild-type yeast (rho+) lifespan compared to high-methionine treatment, rho+ yeast were shorter lived than rho0 yeast when both were subjected to low methionine. Thus, the action of low methionine appears to be opposite that of spermidine in rho0 cells, while being similar to spermidine in rho+ cells. This suggests that mitochondrial function plays a significant role in the ability of both treatments to modulate lifespan. We also observed that spermidine could rescue low-methionine toxicity in rho0 yeast, presumably by providing methionine through the pathway mentioned. We then uncovered that deficiency of CYT1 (a subunit of mitochondrial complex III) alone replicates the effect of complete mtDNA-deficiency with respect to spermidine toxicity. Future work will continue to explore the interaction between spermidine and low-methionine modulation of lifespan with the goal of identifying the molecular targets involved.

